# Supplementary figures and images for: Cellular transcriptomics of arrested normal lung fibroblasts IMR-90 infected with Human Adenovirus 5 E1A mutants
Source: PLoS One. 2025 May 27;20(5):e0323494. doi: 10.1371/journal.pone.0323494 (PMC12112082; doi:10.1371/journal.pone.0323494)

A

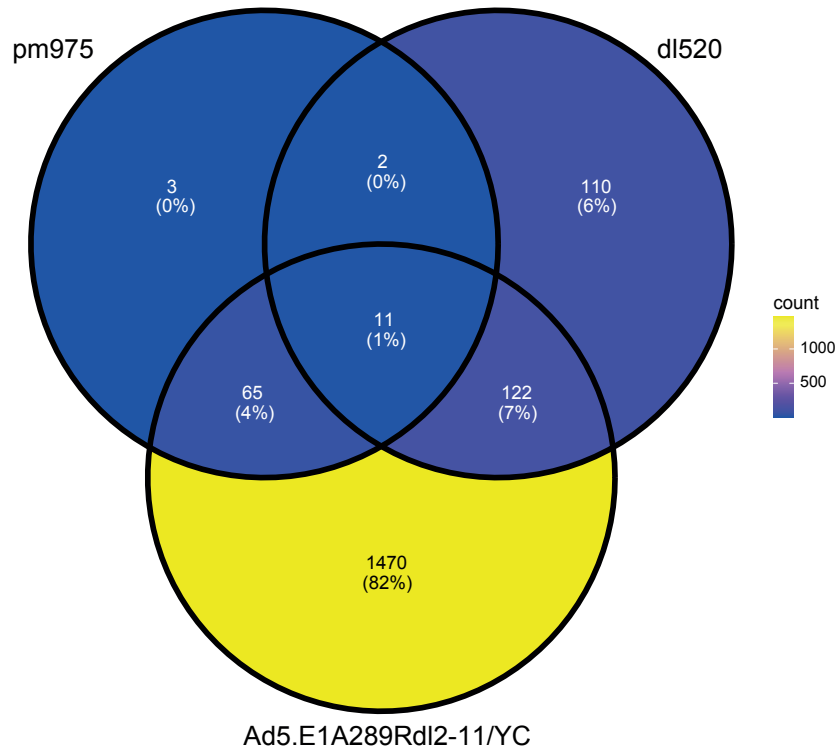

B

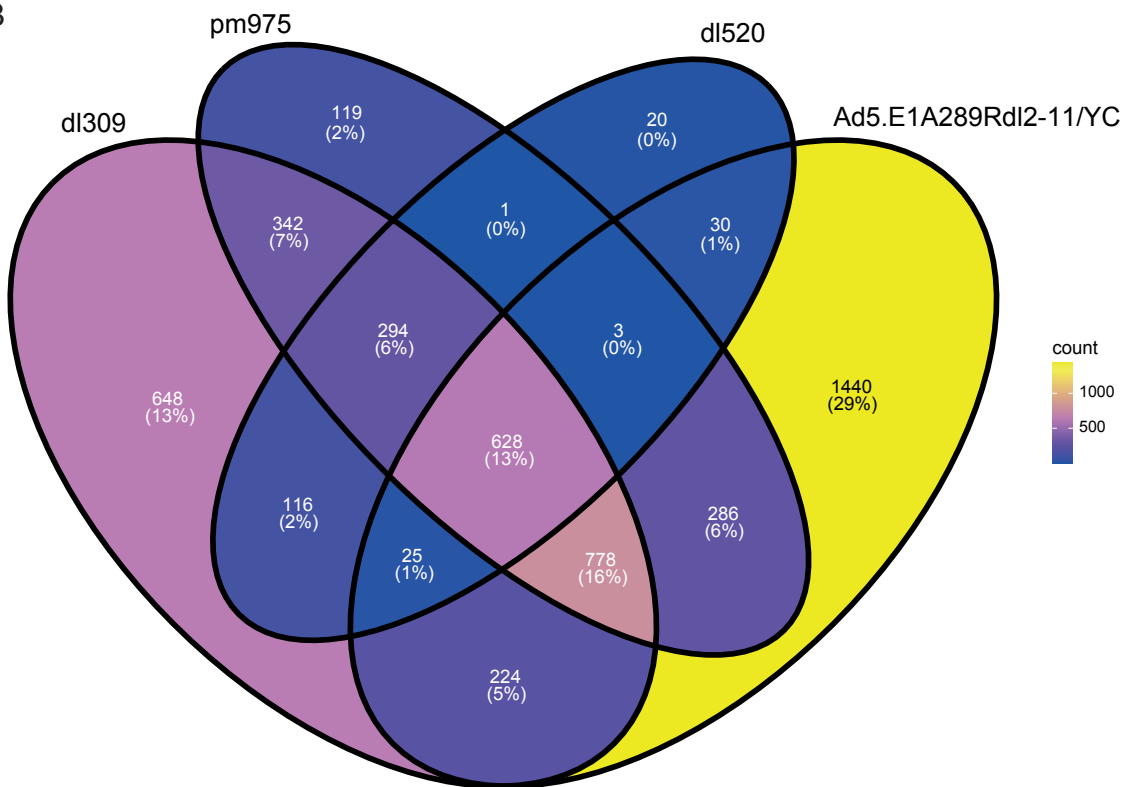

Supplement: Supplemental Fig 1 — (PDF) [file pone.0323494.s001.pdf]

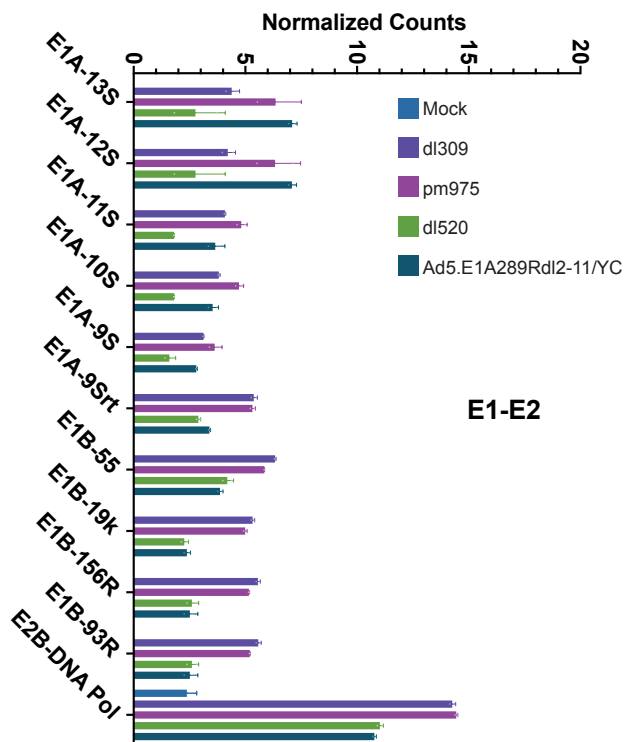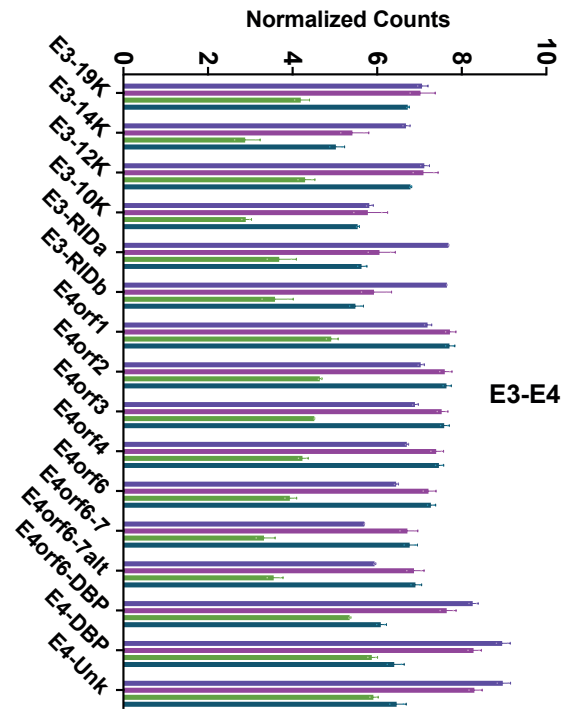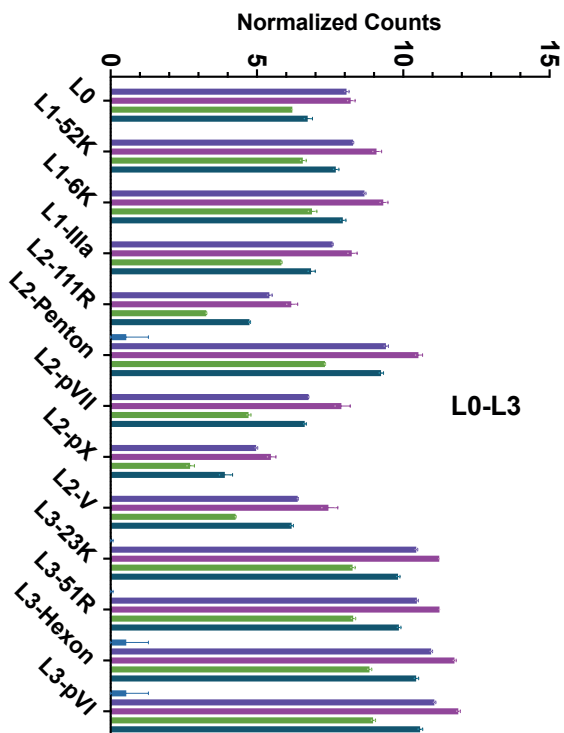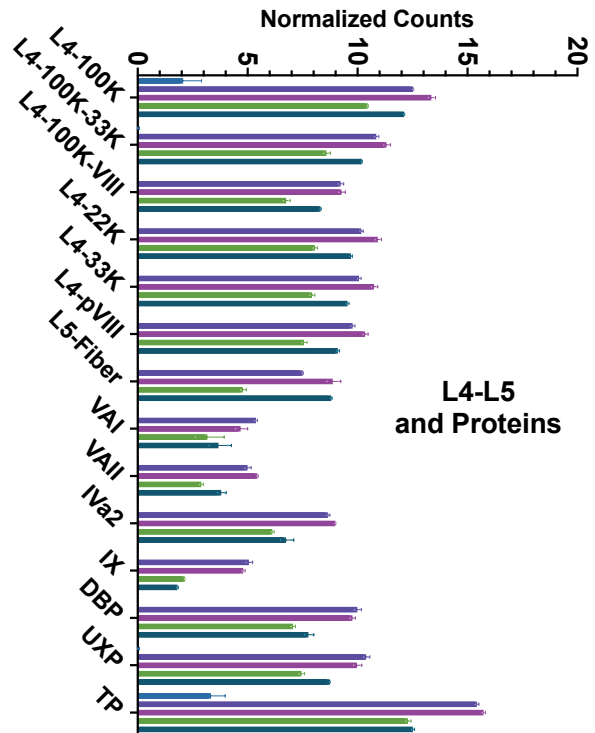

Supplemental Figure 3 - Helwer & Pelka

Supplement: Supplemental Fig 3 — (PDF) [file pone.0323494.s003.pdf]

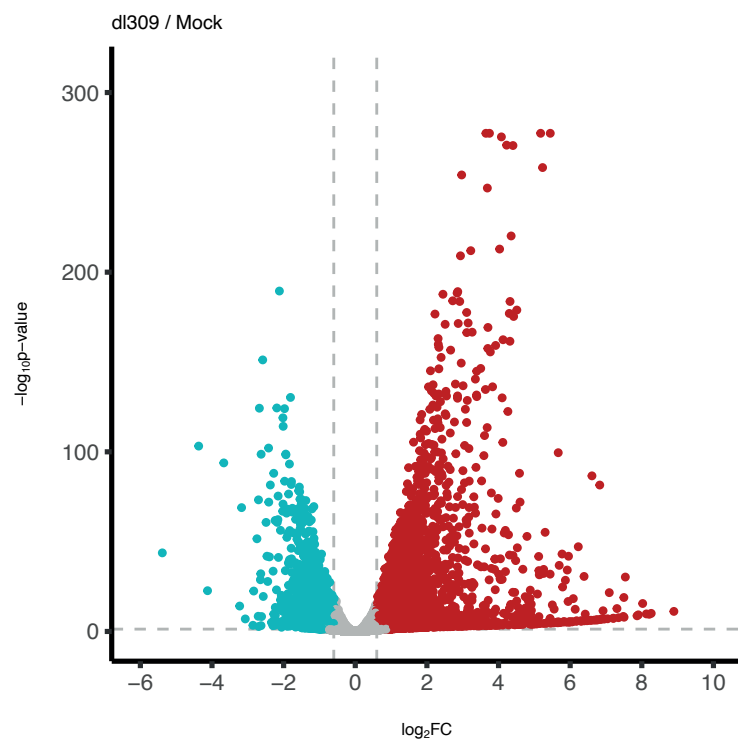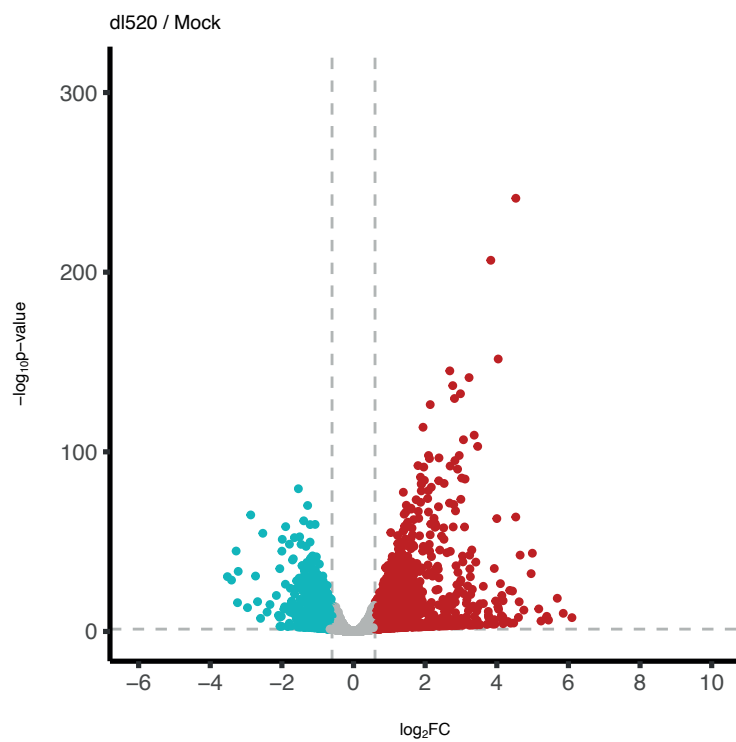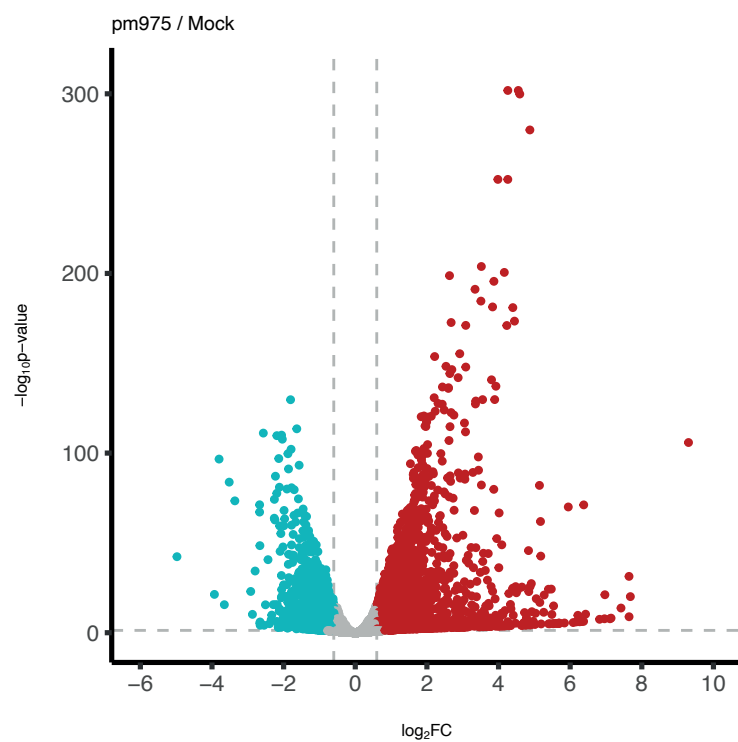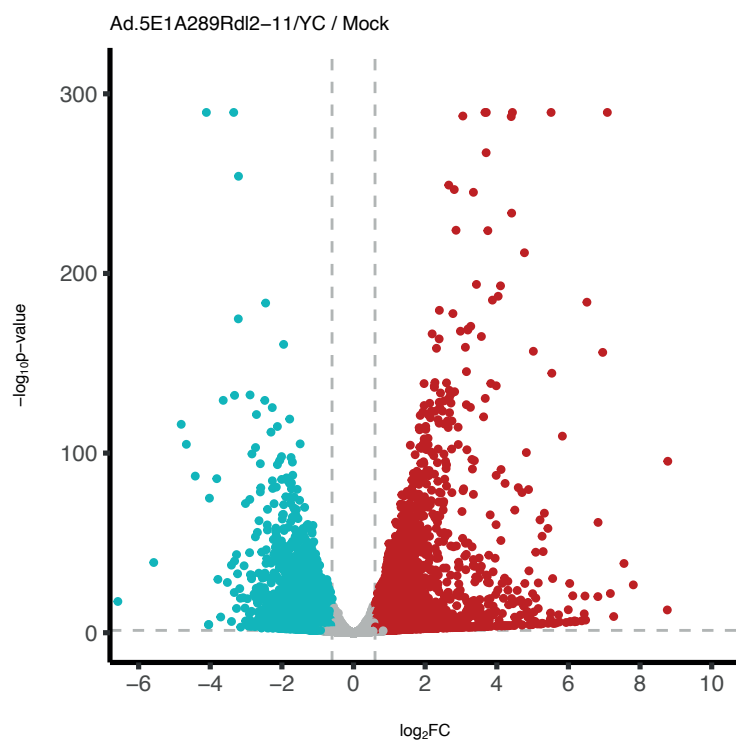

Supplement: Supplemental Fig 4 — (PDF) [file pone.0323494.s004.pdf]

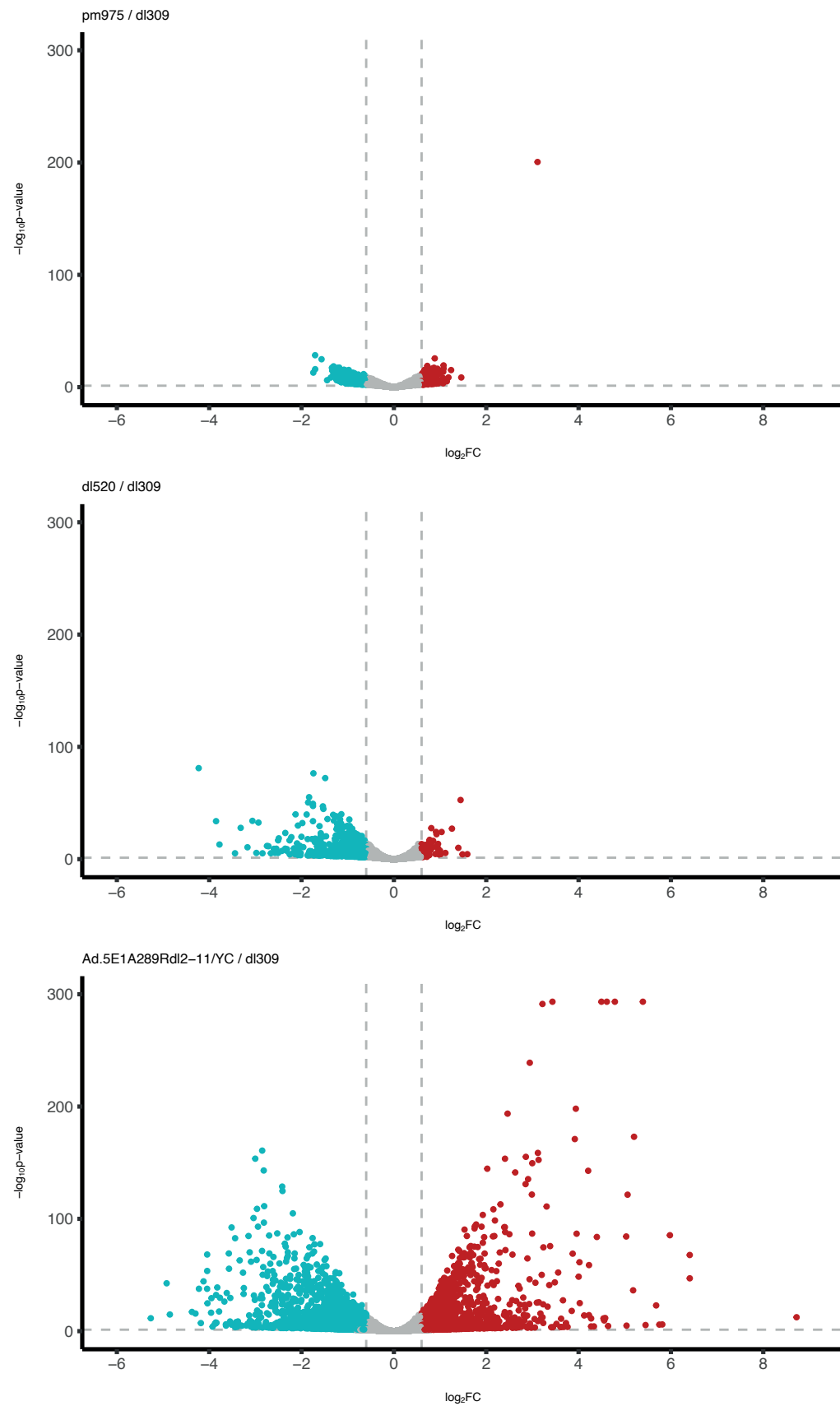

Supplemental Figure 5 - Helwer & Pelka

Supplement: Supplemental Fig 5 — (PDF) [file pone.0323494.s005.pdf]

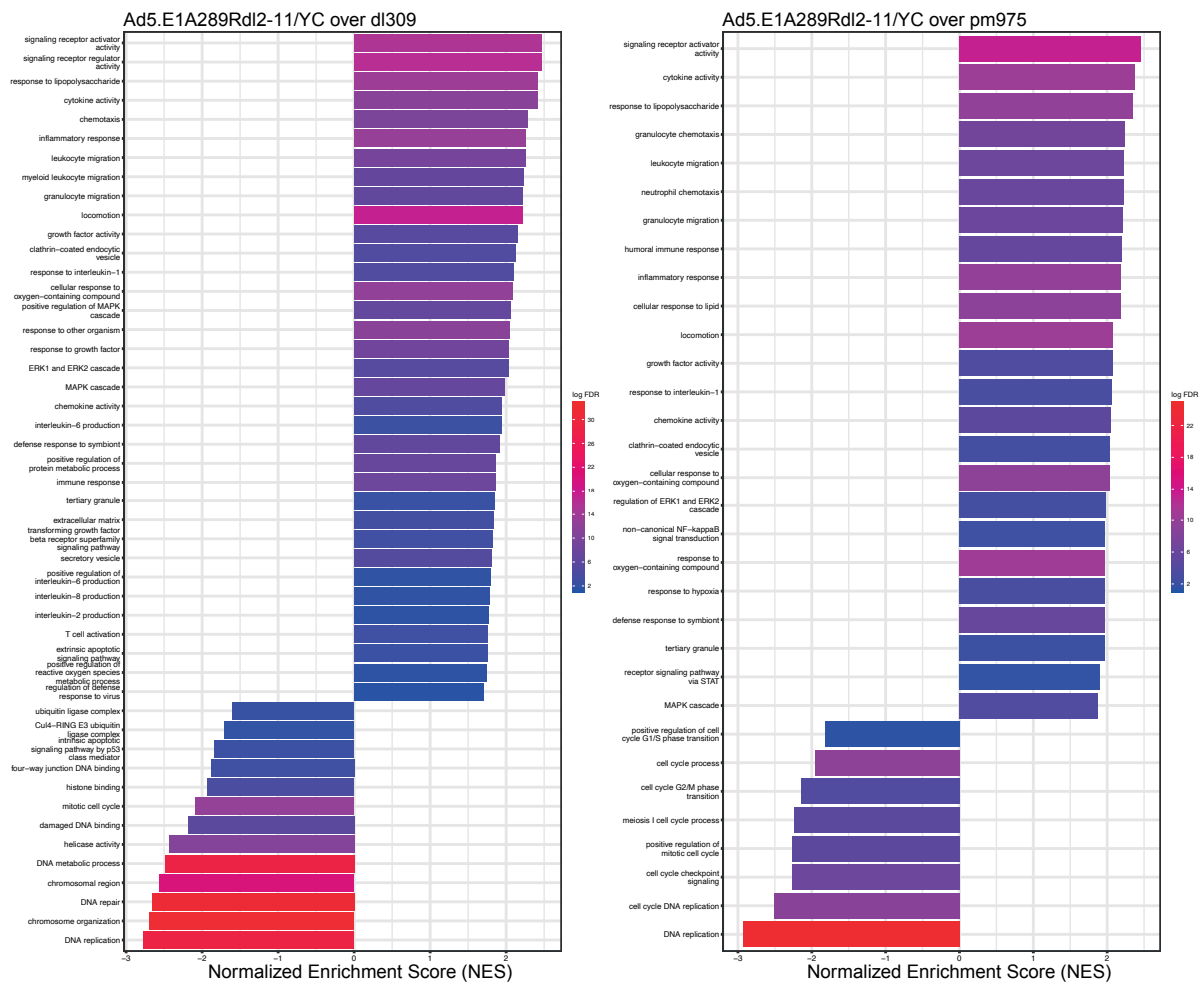

Ad5.E1A289Rdl2-11/YC over dI520

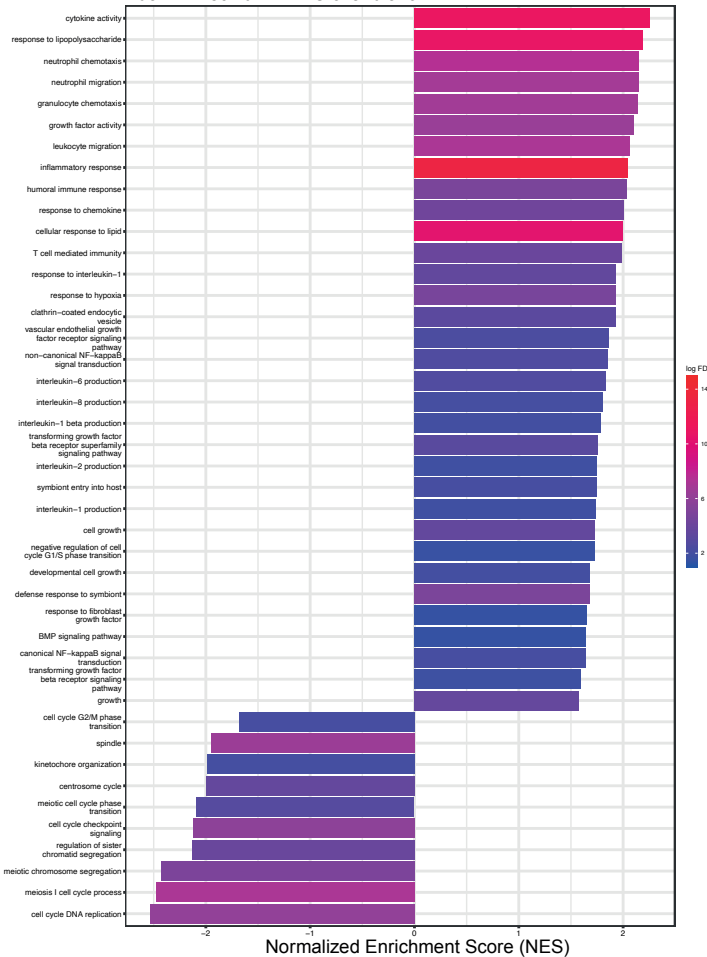

Supplement: Supplemental Fig 6 — (PDF) [file pone.0323494.s006.pdf]
