# Supplementary material for: Cellular transcriptomics of arrested normal lung fibroblasts IMR-90 infected with Human Adenovirus 5 E1A mutants
Source: PLoS One. 2025 May 27;20(5):e0323494. doi: 10.1371/journal.pone.0323494 (PMC12112082; doi:10.1371/journal.pone.0323494)
Supplement: Supplemental Fig 2 — (PDF) [file pone.0323494.s002.pdf]

Differentially Expressed Genes in Ad5.E1A289Rdl2-11/YC over pm975

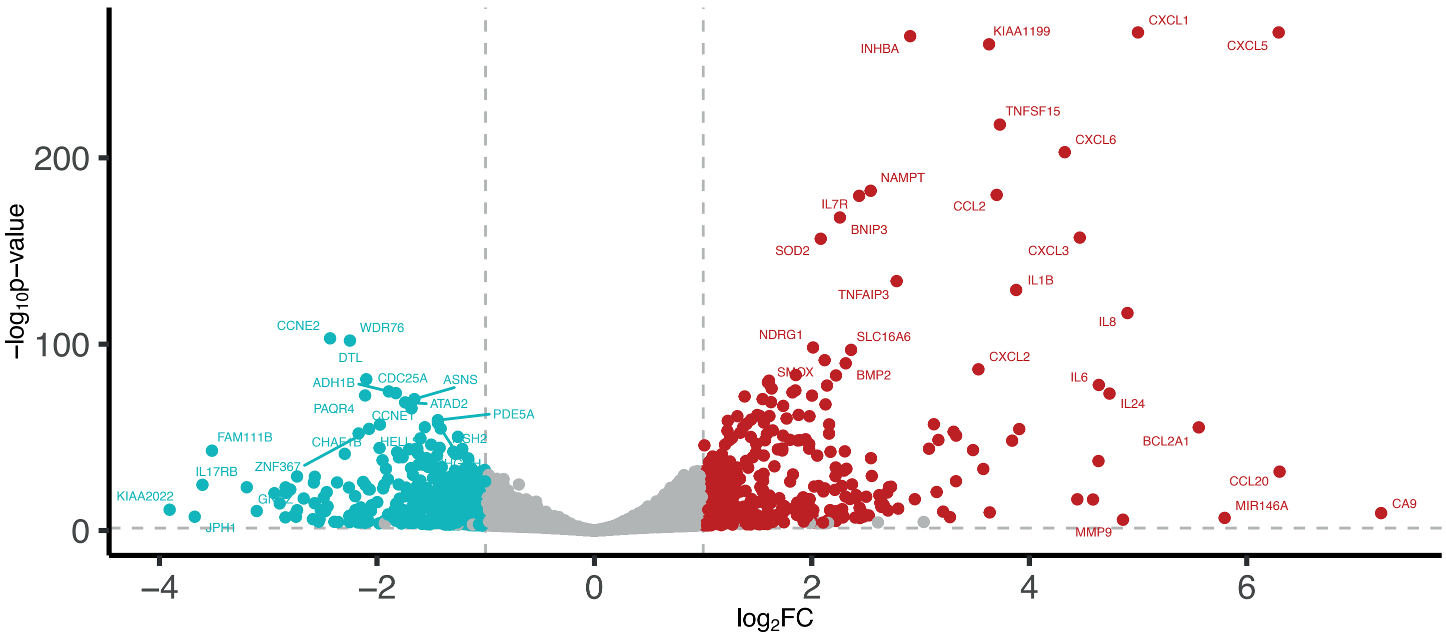

Differentially Expressed Genes in Ad5.E1A289Rdl2-11/YC over dl520

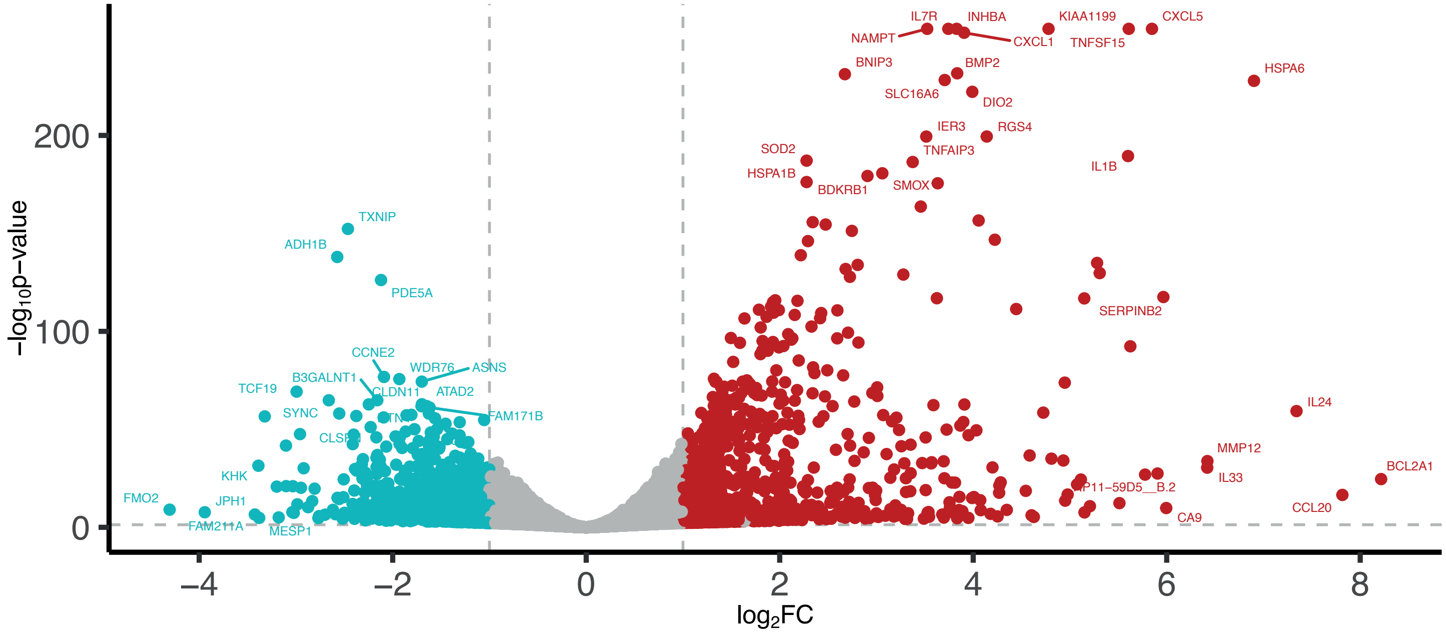

Supplemental Figure 2 - Helwer & Pelka
