# Supplementary material for: Cellular transcriptomics of arrested normal lung fibroblasts IMR-90 infected with Human Adenovirus 5 E1A mutants
Source: PLoS One. 2025 May 27;20(5):e0323494. doi: 10.1371/journal.pone.0323494 (PMC12112082; doi:10.1371/journal.pone.0323494)
Supplement: Attachment — Submitted filename: Rebuttal.pdf [file pone.0323494.s009.pdf]

We wanted to thank the reviewers for their hard work and insightful comments. We have addressed all of the reviewers comments and our point-by-point response is provided below. Original reviewer comments are in *italics*.

Reviewer #1.

"Of particular necessity is the expression levels of the different E1A proteins to ascertain that the different effect seen on transcription are not simply due to big difference in expression levels. It would also be interesting to have expression levels of additional viral proteins, especially the early proteins E4orf6 and E1B55K, as described below in the first point of the minor issues"

Response: We have now included the requested E1A blot as Figure 2A showing similar levels of E1A protein across all viruses. We have attempted to blot for E4orf6 and E1B-55k but were unable to obtain satisfactory results as these antibodies are not as robust as the E1A antibody and expression is expected to be low at 16 hours after infection. Nevertheless, viral gene expression is shown in Supplemental Figure 3 showing comparable levels of these genes mRNAs in the viruses examined.

"In the introduction (line 51), the authors mention other viral protein can contribute to the dysregulation of the cell cycle in infected cells. They have however forgotten to add the ligase complex formed by the E4orf6 protein with the E1A55K as shown by Dallaire et al. 2015 mSphere 1 (1)."

Response: We apologize for the omission, and this reference has now been incorporated into the introduction.

"Line 73 That sentence is confusing, should be reworded."

Response: This has been reworded.

"Fig. 2 (line 143) indicates that significant expression alteration is a change of more than 2 whereas the Materials and Methods section indicates a change of more than 1.5. It should be consistent."

Response: This was changed to be consistent.

"Fig. 4 The fourth panel is not described in the text."

Response: The description has not been added to the results.

"The Material and methods section indicates that viral transcripts were also analysed. It would be interesting to also show and discuss these."

Response: This is now included as Supplemental Figure 3.

Reviewer #2:

"I raise two issues of cosmetic concern regarding the volcano plots in Figs. 4 and 5. First, the font size used to label significant genes is far too small to be of any use in a printed copy of the report. Perhaps fewer genes could be labeled, or the reader can simply refer to the sorted data. Second, in my opinion, the limits of the x axis in Fig. 5 should be made identical. Since I recommend eliminating the labeled genes, by harmonizing the scale between the three panels, the striking absence of an effect for the 12S- and 13S-expressing viruses compared to the 13S-mutant becomes much more obvious."

Response: We have now increased the size of the text labels on the volcano plots (Figures 4 and 5) and have included versions of volcano plots with harmonized Y-axes as suggested by the reviewer as Supplemental Figures 4 and 5.

We hope that the changes we have made will make our manuscript suitable for publication in PLOS One.

Thank you,

The authors.
